# Clinical outcomes of patients with hepatic insufficiency undergoing transcatheter aortic valve implantation: a systematic review and meta-analysis

**DOI:** 10.1186/s12872-022-02510-2

**Published:** 2022-02-23

**Authors:** Wenkai Jiang, Zeyi Cheng, Shiyan Tu, Xing Wang, Caifei Xiang, Wence Zhou, Lin Chen

**Affiliations:** 1 The First Clinical Medical College of Lanzhou University, No.222, Tianshui Road (South), Chengguan District, Lanzhou City, 730000 Gansu Province China; 2 Department of Cardiac Surgery, Ruijin Hospital affiliated to School of Medicine, Shanghai Jiao Tong University, No.197, Ruijin Road (Second), Shanghai City, 200025 China; 3 The Second Clinical Medical College of Lanzhou University, No.222, Tianshui Road (South), Chengguan District, Lanzhou City, 730000 Gansu Province China; 4 Department of General Surgery, The First Hospital of Lanzhou University, No. 1, Donggangxi Road, Chengguan District, Lanzhou City, 730000 Gansu Province China; 5 Department of Infectious Diseases, The First Hospital of Lanzhou University, No. 1, Donggangxi Road, Chengguan District, Lanzhou City, 730000 Gansu Province China

**Keywords:** Transcatheter aortic valve implantation, Surgical aortic valve replacement, Hepatic insufficiency, Meta-analysis, Mortality

## Abstract

**Background:**

Transcatheter aortic valve implantation (TAVI) is currently a common treatment in high-risk aortic stenosis patients, but the impact of hepatic insufficiency on prognosis after TAVI is debatable and whether TAVI is superior to surgical aortic valve replacement (SAVR) in patients with hepatic insufficiency is uncertain.

**Objective:**

To investigate the effect of abnormal liver function on the outcome and safety after TAVI and whether TAVI is superior to SAVR in patients with hepatic insufficiency.

**Methods:**

PubMed, Embase, the Cochrane Library and Web of Science were systematically searched from inception up to 26 November 2021. Studies were eligible if mortality and complications after TAVI in patients with and without hepatic insufficiency, or mortality and complications for TAVI versus SAVR in patients with hepatic insufficiency were reported. The Newcastle–Ottawa scale (NOS) was used to evaluate the quality of each study. This meta-analysis was registered with PROSPERO (CRD42021253423) and was carried out by using RevMan 5.3 and Stata 14.0.

**Results:**

This meta-analysis of 21 studies assessed a total of 222,694 patients. Hepatic insufficiency was associated with higher short-term (in-hospital or 30-day) mortality [OR = 1.62, 95% CI (1.18 to 2.21), *P* = 0.003] and 1–2 years mortality [HR = 1.64, 95% CI (1.42 to 1.89), *P* < 0.00001] after TAVI. Between TAVI and SAVR in patients with hepatic insufficiency, there was a statistically significant difference in in-hospital mortality [OR = 0.46, 95% CI (0.27 to 0.81), *P* = 0.007], the occurrence rate of blood transfusions [OR = 0.29, 95% CI (0.22 to 0.38), *P* < 0.00001] and the occurrence rate of acute kidney injury [OR = 0.55, 95% CI (0.33 to 0.91), *P* = 0.02].

**Conclusions:**

TAVI patients with hepatic insufficiency may have negative impact both on short-term (in-hospital or 30-day) and 1–2-years mortality. For patients with hepatic insufficiency, TAVI could be a better option than SAVR.

**Supplementary Information:**

The online version contains supplementary material available at 10.1186/s12872-022-02510-2.

## Introduction

Transcatheter aortic valve implantation (TAVI) is currently a common treatment in high-risk aortic stenosis patients [1–3]. Since the US Food and Drug Administration approval in 2011, the safety of TAVI has been continuously improved and its indications have been expanded [4]. However, TAVI patients tend to have particularly high-risk baseline profiles, and the number of surviving patients falls off rapidly over time [1, 5, 6]. The impact of hepatic insufficiency on prognosis after TAVI is debatable. For example, hyperbilirubinemia and hypoproteinemia, will lead to higher mortality and stroke rates after TAVI, but some studies have shown that there is no significant increase in complications after TAVI for liver transplantation patients [7–9]. We conducted a systematic review and meta-analysis of existing studies to assess whether hepatic insufficiency is associated with increased mortality after TAVI and whether TAVI is superior to surgical aortic valve replacement (SAVR) in patients with hepatic insufficiency, aiming to provide ideas for improving the prognosis of patients with aortic valve disease or patients undergoing TAVI.

## Methods

### Protocol and guidance

This study was performed in accordance with the Preferred Reporting Items for Systematic Reviews and Meta-Analysis (PRISMA) [10]. The protocol for this systematic review and meta-analysis was registered with PROSPERO (CRD42021253423).

### Search strategy

Two of the authors (WJ and CX) conducted the search of several databases: PubMed, Embase, the Cochrane Library, and Web of Science, by 26 November 2021. We used the following MeSH terms and/or free-text terms: “hepatic insufficiency”, “aortic valve stenosis”, and “transcatheter aortic valve replacement”. Additional file 1: Table S1 presents the search strategy of PubMed.

### Inclusion and exclusion criteria

Two analyses will be presented in the paper. The first is the influence of hepatic insufficiency or not on the postoperative outcome of TAVI. The second is the outcome comparison between TAVI and SAVR in patients with hepatic insufficiency.

The studies were eligible to access the influence of hepatic insufficiency or not on the postoperative outcome of TAVI according to the following inclusion criteria: (1) population: patients undergoing TAVI; (2) intervention and comparison: compare the patients with hepatic insufficiency (impaired liver function and abnormal imaging findings by any causes of liver cirrhosis and liver diseases) or not with; (3) outcomes: the primary outcome was all cause mortality, including short-term mortality (in-hospital and 30-day mortality), 1 year mortality and 2 years mortality, and secondary outcomes were postoperative complications. We considered trials to be eligible to compare the clinical outcome between TAVI and SAVR in patients with hepatic insufficiency according to the following inclusion criteria: (1) population: patients with hepatic insufficiency; (2) intervention and comparison: compared TAVI with SAVR; (3) outcomes: the primary outcome was all cause mortality, including short-term mortality (in-hospital and 30-day mortality), 1 year mortality and 2 years mortality, and secondary outcomes were postoperative complications.

We excluded studies if they were conference proceedings, guidelines, systematic reviews, case reports, letters and studies without full-text literature; if hazard ratios (HR), odds ratios (OR) and their 95% confidence intervals (CI), or sufficient raw data could not be obtained or calculated.

### Study selection and data extraction

Two independent researchers (WJ and ZC) screened all titles and abstracts and reviewed full texts when studies were deemed eligible. Then, two researchers (WJ and ZC) independently performed the data extraction process using a standard data extraction form to extract data from the included studies. Disagreements were resolved by consensus.

### Risk of bias and quality assessment

The Newcastle–Ottawa scale (NOS) was used to evaluate the quality of each study [11]. The NOS used for cohort studies consists of three categories: selection, comparability, and outcome. A study can be awarded from zero up to nine stars. The certainty of the overall evidence was assessed following the Grading of Recommendations, Assessment, Development and Evaluation (GRADE) approach. Publication bias was assessed with funnel plots for asymmetry using Egger’s tests [12].

### Statistical analysis

To pool study results, this meta-analysis was carried out by using random effects models in RevMan 5.3 and Stata 14.0. We used HRs and ORs and their associated 95% CIs to assess outcomes, and considered a *P* value less than 0.05 to be statistically significant. Heterogeneity was assessed by calculating the I^2^ statistic and its *P* value [13].

## Results

### Study selections and study characteristics

**Fig. 1:**
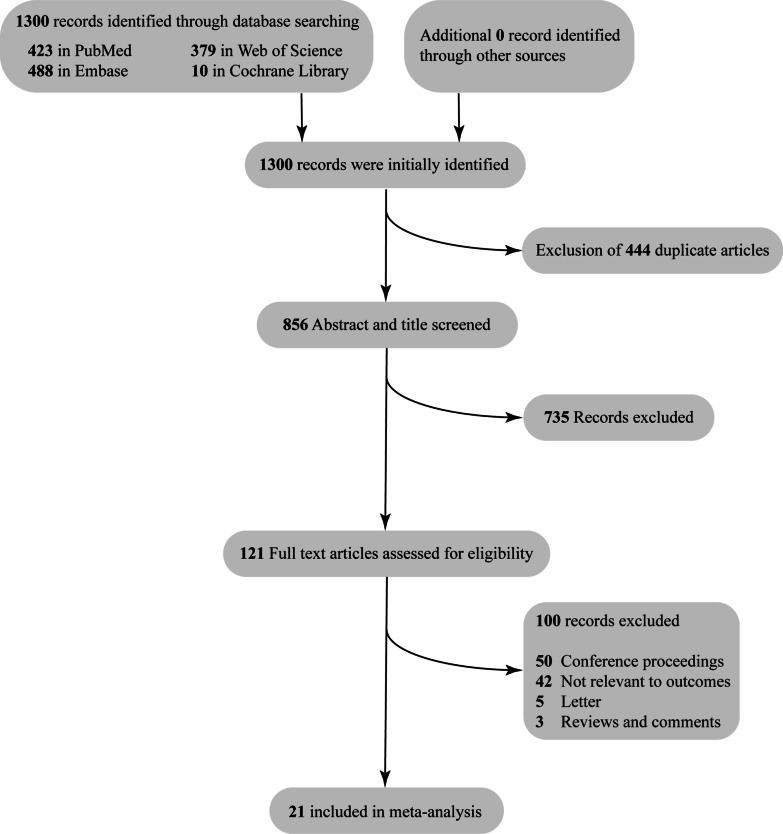
Flow chart of study selection of this meta-analysis

A total of 1300 records were initially identified. After exclusion of 444 duplicate articles, the remaining articles underwent title and abstract review. 735 articles were excluded at this stage since they were not related to this meta-analysis, leaving 121 articles for full-length article review. Therefore, 21 studies were finally included in this meta-analysis (14 were used to analyse the influence of hepatic insufficiency or not on the postoperative outcome of TAVI and 7 were used to analyse the outcome comparison between TAVI and SAVR in patients with hepatic insufficiency). The flow chart of study selection is shown in Fig. 1. Summaries of the included studies and the clinical characteristics are shown in Tables 1 and 2.

**Table 1:** Patients baseline characteristics in the first analysis

Study	Patient number	Age	Male (%)	LD (%)	Country	History of PCI (%)	History of CABG (%)
Beohar 2014	485	NR	53.80	2.7	American	NR	NR
Beohar 2016	1063	84.67	56.50	2.6	American	NR	36.7
Elbadawi 2019	20,210	80.79	51.3	2.6	American	18.5	20.5
Krittanawong 2020	6368	81.4 ± 8.1	50.4	2.5	American	8.6	0.5
Lantelme 2020	20,443	82.7	50.0	5.8	France	NR	NR
Lee 2021	2424	NR	57.6	25	American	NR	0.5
Schymik 2015	2688	81.4±6.6	42.3	2.9	17 countries	30.5	16.0
Thomas 2011	1038	81.1	44.5	3.0	Europe	NR	22.7
Thourani 2016	2531	84.4	52.4	2.7	American	39.6	42.6
Tirado-Conte 2018	228	75.2	61.4	NR	Europe and Canada	NR	9.2
Ullah 2020	161,049	NR	53.4	2.0	American	6.2	17.7
Wendler 2013	1387	80.6 ± 7.1	41.5	3.4	Europe	28.5	25.5
Wendt 2017	640	80.4	43.8	NR	Germany	NR	NR
Yassin 2018	226	79.5	45.0	NR	American	NR	NR

*LD* liver disease, *NR* not reported

**Table 2:** Patients baseline characteristics in the second analysis

Study	Patient number	Age	Male	Country
*Alqahtani 2017*				
TAVR	134	71 ± 11	62.7%	
SAVR	134	71 ± 10	59.0%	America
*P*	–	0.784	0.641	
*Dhoble 2017*				
TAVR	55	67.2	65.5%	America
SAVR	55	67.0	65.5%	
*P*	–	0.893	1.00	
*Greason 2013*				
TAVR	6	NR	83.0%	America
SAVR	12	NR	83.0%	
*P*	–	–	0.73	
*Khan 2020*				
TAVR	298	67.4 ± 8.6	71.6%	America
SAVR	901	65.7 ± 9.3	71.6%	
*P*	–	< 0.01	0.98	
*Peeraphatdit 2020*				
TAVR	55	75.4 ± 9.4	70.9%	America
SAVR	50	68.4 ± 8.7	64.0%	
*P*	–	0.0002	0.45	
*Seppelt 2020*				
TAVR	43	75.2	62.8%	Germany
SAVR	42	71.6	47.6%	
*P*	–	NR	NR	
*Thakkar 2015*				
TAVR	36	73.36 ± 1.69	77.8%	America
SAVR	93	66.08 ± 0.89	67.7%	
*P*	–	< 0.0001	0.3	

*NR* not reported, *TAVI* transcatheter aortic valve implantation, *SAVR* surgical aortic valve replacement

### Study quality

All included studies that underwent quality assessment with the use of the NOS received a total of 6 to 8 stars, and were thus deemed to have a low risk of bias. The results of the study quality are shown in Additional file 1: Table S2 and S3. The GRADE quality assessment of all outcomes is shown in Additional file 1: Table S4.

### Results of meta-analysis

Fourteen studies were identified to analyse the influence of hepatic insufficiency or not on the outcome of TAVI. Eight studies [14–20] were included in the analysis of 1–2 years mortality, all of which reported the HR value of hepatic insufficiency (compared with no hepatic insufficiency) in Cox survival analysis. Seven studies [21–27] were included in the short-term mortality analysis.

#### In-hospital and 30-day mortalities

**Fig. 2:**
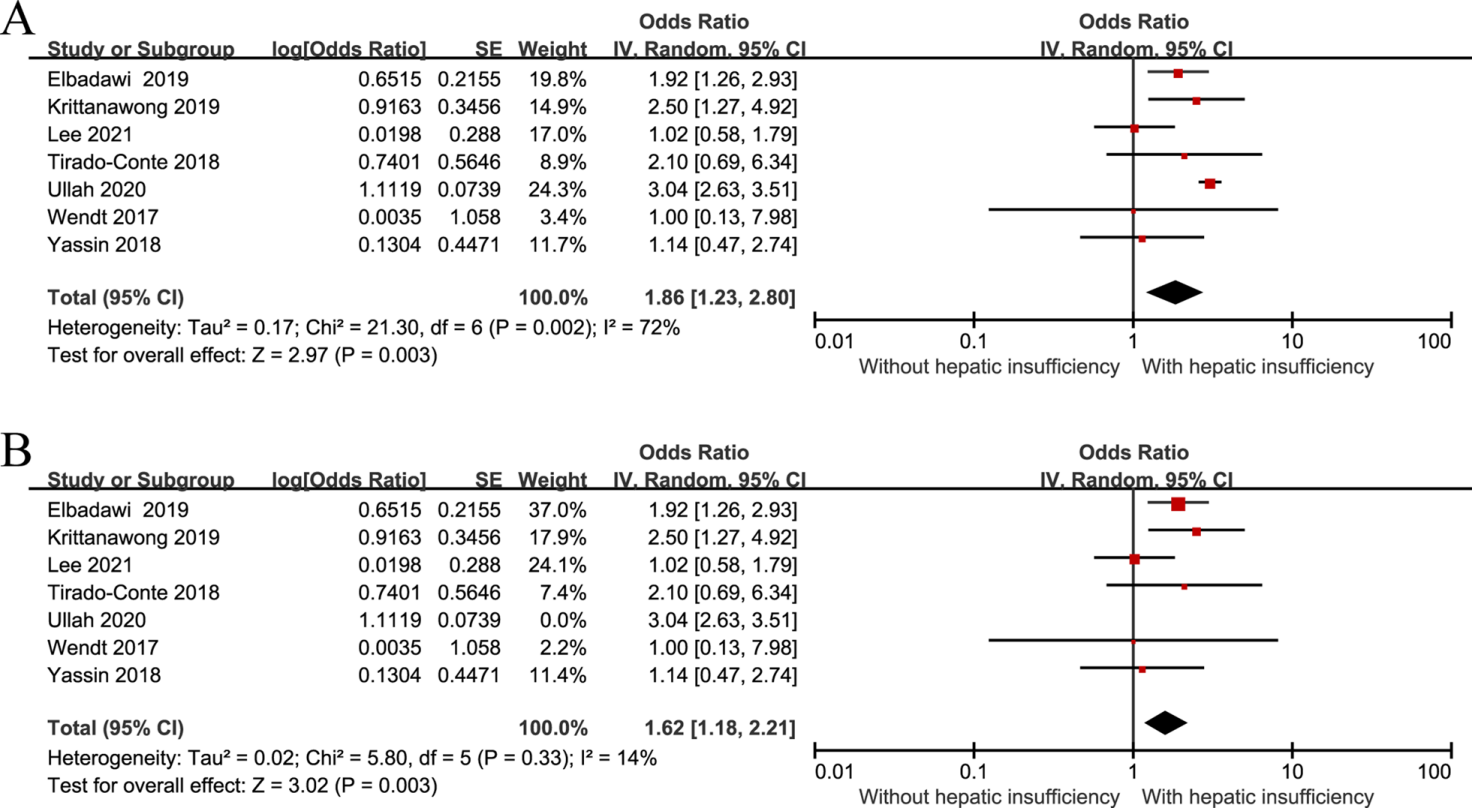
Forest plot of short-term mortality after TAVI in patients with and without hepatic insufficiency (**A** 7 trials were included, and there was heterogeneity; **B** Ullah 2020 was excluded)

In this meta-analysis, hepatic insufficiency showed a detrimental effect on short-term mortality after TAVI [OR = 1.86, 95% CI (1.23–2.80), *P* = 0.003; *P* for heterogeneity = 0.002, I^2^ = 72%, Fig. 2A]. Sensitivity analysis was performed to evaluate the impact of qualitative heterogeneity on the pooled effect estimate. Individual studies were excluded one by one, and heterogeneity decreased when Ullah 2020 [24] was excluded (*P* for heterogeneity = 0.33, I^2^ = 14%, Fig. 2B), suggesting that heterogeneity was caused by Ullah 2020. The final meta-analysis results showed that patients with hepatic insufficiency were at a 1.88-fold higher risk than patients without hepatic insufficiency [OR = 1.62, 95% CI (1.18 to 2.21), *P* = 0.003; *P* for heterogeneity = 0.33, I^2^ = 14%, Fig. 2B].

#### 1–2 years mortality

**Fig. 3:**
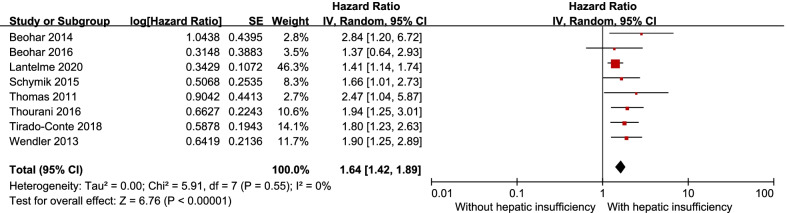
Forest plot of 1–2 years mortality TAVI in patients with and without hepatic insufficiency

Eight studies compared 1–2 years mortality between patients with hepatic insufficiency and without hepatic insufficiency after TAVI. The results showed that hepatic insufficiency was associated with higher 1–2 years mortality [HR** = **1.64, 95% CI (1.42–1.89), *P* < 0.00001; *P* for heterogeneity = 0.55, I^2^ = 0%, Fig. 3] and there was statistically significant funnel plot asymmetry (two-tailed *P* = 0.032).

When focusing on the outcome between TAVI and SAVR in patients with hepatic insufficiency, 7 studies [28–34] were included, and all of them reported the number of outcome events (number of hospitalized deaths, number of blood transfusions and number of acute kidney injury) and the total number of people in both the hepatic insufficiency group and the non-hepatic insufficiency group. Adjusted data were extracted from propensity score matching (PSM) pairs in 4 studies, and unadjusted data were abstracted from 3 studies.

#### In-hospital mortality

**Fig. 4:**
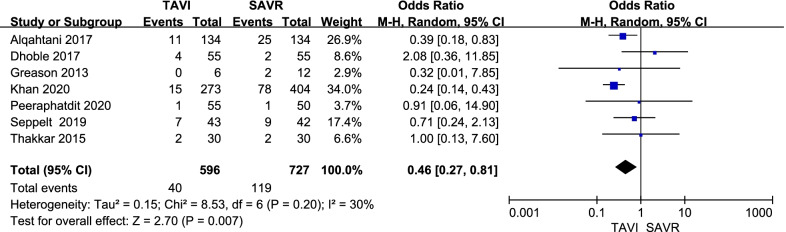
Forest plot of in-hospital mortality after TAVI versus SAVR in patients with hepatic insufficiency

Seven studies compared in-hospital mortality between the two groups. The pooled analysis indicated that there was a statistically significant difference in in-hospital mortality between TAVI and SAVR in patients with hepatic insufficiency [OR = 0.46, 95% CI (0.27–0.81), *P* = 0.007; *P* for heterogeneity = 0.20, I^2^ = 30%, Fig. 4] and no statistically significant funnel plot asymmetry (two-tailed *P* = 0.263).

#### Secondary outcomes: postoperative complications

**Fig. 5:**
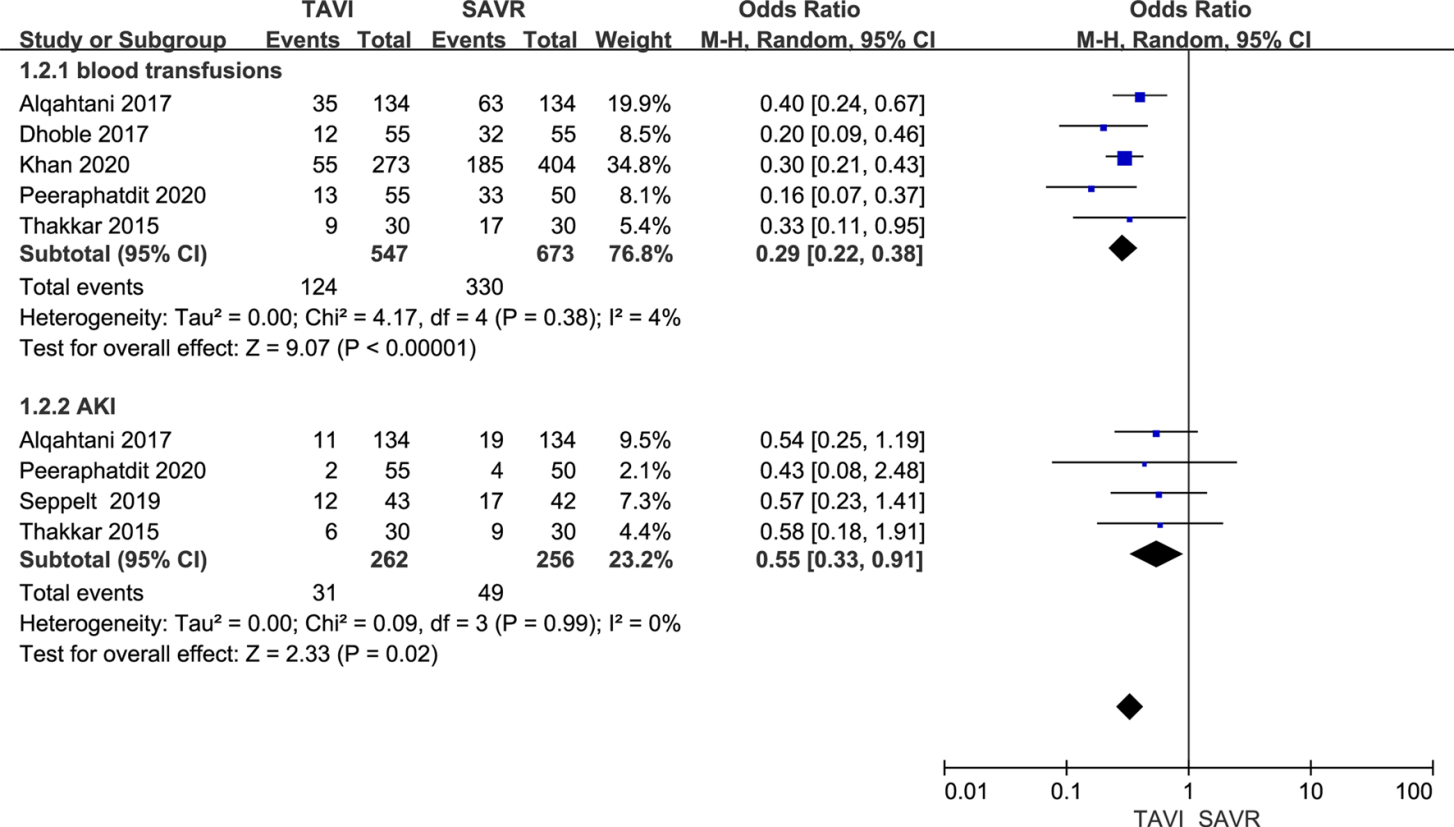
Forest plot of complications after TAVI versus SAVR in patients with hepatic insufficiency

In addition, postoperative complications after TAVI or SAVR were also evaluated. The occurrence rate of blood transfusions was evaluated in five studies. Heterogeneity among trials was not significant and we found a  significant difference between the two groups [OR = 0.29, 95% CI (0.22–0.38), *P* < 0.00001; *P* for heterogeneity = 0.38, I^2^ = 4%]. Four trials reported the occurrence rate of acute kidney injury (AKI). Heterogeneity among trials was not significant, and we found a significant difference between the two groups [OR = 0.55, 95% CI (0.33–0.91), *P* = 0.02; *P* for heterogeneity = 0.99, I^2^ = 0%] (Fig. 5).

## Discussion

The main results of the present meta-analysis are as follows: TAVI patients with hepatic insufficiency may have negative impact both on short-term (in-hospital or 30 days) and 1–2-years mortality; among patients with hepatic insufficiency, there was a statistically difference in in-hospital mortality after TAVI and SAVR, and SAVR was more likely to have AKI and blood transfusion.

There is a high proportion in TAVI patients with coronary artery disease, chronic obstructive pulmonary disease and chronic kidney disease, and these diseases are also risk factors for high postoperative mortality and a high occurrence rate of complications [5, 6, 35]. The low prevalence of hepatic insufficiency in TAVI patients does not mean that these patients do not have to be evaluated preoperatively. This meta-analysis evaluated the impact of hepatic insufficiency on postoperative mortality after TAVI, and the results showed that hepatic insufficiency could lead to increased short-term and 1–2 years mortality after TAVI.

There are limited studies that compare the postoperative conditions of patients with and without hepatic insufficiency. Tirado-Conte et al. [23] compared the incidence of postoperative complications in patients with and without hepatic insufficiency. The results showed that patients with hepatic insufficiency were more likely to have stage 1 AKI after TAVI (23% vs. 9%, *P* = 0.011), and had longer hospital stays [median 7 (6–11) vs. median 6 (5–8), *P* = 0.017]. In the study by Yassin et al. [26], hepatic insufficiency affected the incidence of acute myocardial infarction after TAVI (4.45% vs. 2.78% without hepatic insufficiency), but the difference was not statistically significant. More studies are needed to focus on the incidence of postoperative complications in patients with hepatic insufficiency after TAVI.

Cardiac surgical teams should consider whether to perform TAVI or SAVR in cases of severe comorbidities. Studies have shown that compared with SAVR, patients undergoing TAVI have lower mortality and fewer complications [36–38]. The results of our meta-analysis showed that TAVI patients with hepatic insufficiency had lower in-hospital mortality than SAVR and fewer postoperative complications (blood transfusion and AKI). In addition, other outcomes after TAVI were superior to SAVR. For example, in the 30 pairs of the study by Thakkar et al. [34], the postprocedural length of stay in the TAVI group was shorter than that in SAVR group (6.2 days vs. 14.3 days, *P* = 0.006). In the study by Alqahtani et al. [28], the non-family discharge rate in the SAVR group was significantly higher than that in the TAVI group (31.3% vs. 53%, *P* < 0.0001). Therefore, intervention options should be selected after careful, individualized evaluation of the suitability and risks of the technique in high-risk patients (such as those with hepatic insufficiency).

## Limitations

The limitations of our meta-analysis are as follows: Firstly, significant heterogeneity was encountered perhaps due to various operation details, different causes and duration of liver disease, center settings, and populations enrolled, calling for cautious interpretation of the results. Next, all the studies were retrospective studies and may suffer from sources of bias. Moreover, the effect was assessed by a few studies, so the evidence to support it is low, and data related to postoperative complications and hospitalization were not combined, and the severity of hepatic insufficiency was not classified, leading to fewer outcome indicators.

## Conclusions

Overall, TAVI patients with hepatic insufficiency may have negative impact both on short-term (in-hospital or 30-day) and 1–2-years mortality. For patients with hepatic insufficiency, TAVI could be a better option than SAVR. The presence of hepatic insufficiency provides prognostic information that should be taken into account for patients undergoing TAVI.

## Supplementary Information

**Additional file 1.** **Table S1.** PubMed search strategy. **Table S2.** Study quality of the included studies (influence of hepatic insufficiency on the postoperative outcome of TAVI). **Table S3.** Study quality of the included studies (patients with hepatic insufficiency undergoing TAVI verus SAVR). **Table S4.** GRADE quality assessment of all outcomes. **Table S5.** The original data of in-hospital mortality after TAVI versus SAVR in patients with hepatic insufficiency. **Table S6.** The original data of the occurrence rate of blood transfusions after TAVI versus SAVR in patients with hepatic insufficiency. **Table S7.** The original data of the occurrence rate of acute kidney injury after TAVI versus SAVR in patients with hepatic insufficiency.

## Data Availability

All data generated or analysed during this study are included in this article and its Additional file 1 (Figs. 2, 3, 4, 5, Additional file 1: Tables S5, S6, S7)

## Abbreviations

TAVI: Transcatheter aortic valve implantation
SAVR: Surgical aortic valve replacement
HR: Hazard ratio
OR: Odds ratio
CI: Confidence interval
AKI: Acute kidney injury
PSM: Propensity score matching

## References

[1] Didier R, Eltchaninoff H, Donzeau-Gouge P (2018). Five-year clinical outcome and valve durability after transcatheter aortic valve replacement in high-risk patients. Circulation.

[2] Lanz J, Kim WK, Walther T (2019). Safety and efficacy of a self-expanding versus a balloon-expandable bioprosthesis for transcatheter aortic valve replacement in patients with symptomatic severe aortic stenosis: a randomised non-inferiority trial. Lancet.

[3] Vemulapalli S, Carroll JD, Mack MJ (2019). Procedural volume and outcomes for transcatheter aortic-valve replacement. N Engl J Med.

[4] Makkar RR, Yoon SH, Leon MB (2019). Association between transcatheter aortic valve replacement for bicuspid vs tricuspid aortic stenosis and mortality or stroke. JAMA.

[5] Liao YB, He ZX, Zhao ZG (2016). The relationship between chronic obstructive pulmonary disease and transcatheter aortic valve implantation--a systematic review and meta-analysis. Catheter Cardiovasc Interv.

[6] Rattanawong P, Kanitsoraphan C, Kewcharoen J (2019). Chronic kidney disease is associated with increased mortality and procedural complications in transcatheter aortic valve replacement: a systematic review and meta-analysis. Catheter Cardiovasc Interv.

[7] Jou S, Patel H, Oglat H (2020). The prevalence and prognostic implications of pre-procedural hyperbilirubinemia in patients undergoing transcatheter aortic valve replacement. Heart Vessels.

[8] Yamamoto M, Shimura T, Kano S (2017). Prognostic value of hypoalbuminemia after transcatheter aortic valve implantation (from the Japanese Multicenter OCEAN-TAVI Registry). Am J Cardiol.

[9] Ullah W, Sattar Y, Al-Khadra Y (2021). Clinical outcomes of renal and liver transplant patients undergoing transcatheter aortic valve replacement: analysis of national inpatient sample database. Expert Rev Cardiovasc Ther.

[10] Liberati A, Altman DG, Tetzlaff J (2009). The PRISMA statement for reporting systematic reviews and meta-analyses of studies that evaluate health care interventions: explanation and elaboration. PLoS Med.

[11] Stang A (2010). Critical evaluation of the Newcastle–Ottawa scale for the assessment of the quality of nonrandomized studies in meta-analyses. Eur J Epidemiol.

[12] Egger M, Smith GD, Schneider M (1997). Bias in meta-analysis detected by a simple, graphical test. BMJ.

[13] Higgins JP, Thompson SG (2002). Quantifying heterogeneity in a meta-analysis. Stat Med.

[14] Beohar N, Zajarias A, Thourani VH (2014). Analysis of early out-of hospital mortality after transcatheter aortic valve implantation among patients with aortic stenosis successfully discharged from the hospital and alive at 30 days (from the Placement of Aortic Transcatheter Valves Trial). Am J Cardiol.

[15] Beohar N, Kirtane AJ, Blackstone E (2016). Trends in complications and outcomes of patients undergoing transfemoral transcatheter aortic valve replacement: experience from the PARTNER continued access registry. JACC Cardiovasc Interv.

[16] Lantelme P, Lacour T, Bisson A (2020). Futility risk model for predicting outcome after transcatheter aortic valve implantation. Am J Cardiol.

[17] Schymik G, Lefèvre T, Bartorelli AL (2015). European experience with the second-generation edwards SAPIEN XT transcatheter heart valve in patients with severe aortic stenosis: 1-year outcomes from the SOURCE XT registry. J Am Coll Cardiol Interv.

[18] Thomas M, Schymik G, Walther T (2011). One-year outcomes of cohort 1 in the Edwards SAPIEN Aortic Bioprosthesis European Outcome (SOURCE) Registry: the European registry of transcatheter aortic valve implantation using the Edwards SAPIEN valve. Circulation.

[19] Thourani VH, Forcillo J, Beohar N (2016). Impact of preoperative chronic kidney disease in 2,531 high-risk and inoperable patients undergoing transcatheter aortic valve replacement in the PARTNER trial. Ann Thorac Surg.

[20] Wendler O, Waltherb T, Schroefelc H (2013). Transapical aortic valve implantation: mid-term outcome from the SOURCE registry. Eur J Cardio-Thorac Surg.

[21] Elbadawi A, Elgendy IY, Mentias A (2019). Outcomes of urgent versus nonurgent transcatheter aortic valve replacement. Catheter Cardiovasc Interv.

[22] Krittanawon C, Kumar A, Wang Z (2020). Predictors of in-hospital mortality after transcatheter aortic valve implantation. Catheter Cardiovasc Interv.

[23] Tirado-Conte G, Rodés-Cabau J, Rodríguez-Olivares R (2018). Clinical outcomes and prognosis markers of patients with liver disease undergoing transcatheter aortic valve replacement: a propensity score-matched analysis. Circ Cardiovasc Interv.

[24] Ullah W, Zahid S, Hamzeh I (2021). Trends and predictors of transcatheter aortic valve implantation related in-hospital mortality (from the national inpatient sample database). Am J Cardiol.

[25] Wendt D, Kahlert P, Canbay A (2017). Impact of liver indicators on clinical outcome in patients undergoing transcatheter aortic valve implantation. Ann Thorac Surg.

[26] Yassin AS, Subahi A, Abubakar H (2018). Outcomes and effects of hepatic cirrhosis in patients undergoing transcatheter aortic valve implantation. Am J Cardiol.

[27] Lee DU, Han J, Fan GH (2021). The clinical impact of chronic liver disease in patients undergoing transcatheter and surgical aortic valve replacement: systematic analysis of the 2011–2017 US hospital database. Catheter Cardiovasc Interv.

[28] Alqahtani F, Aljohani S, Ghabra A (2017). Outcomes of transcatheter vs. surgical aortic valve implantation for aortic stenosis in patients with hepatic cirrhosis. Am J Cardiol.

[29] Dhoble A, Bhise V, Nevah MI (2018). Outcomes and readmissions after transcatheter and surgical aortic valve replacement in patients with cirrhosis: a propensity matched analysis. Catheter Cardiovasc Interv.

[30] Greason KL, Mathew V, Wiesner RH (2013). Transcatheter aortic valve replacement in patients with cirrhosis. J Card Surg.

[31] Khan MZ, Khan MU, Munir MB (2020). Contemporary trends and outcomes in aortic valve replacement in patients with end-stage liver disease. Catheter Cardiovasc Interv.

[32] Peeraphatdit TB, Nkomo VT, Naksuk N (2020). Long-term outcomes after transcatheter and surgical aortic valve replacement in patients with cirrhosis: a guide for the hepatologist. Hepatology.

[33] Seppelt PC, Zappel J, Weiler H (2020). Aortic valve replacement in patients with preexisting liver disease: transfemoral approach with favorable survival. Catheter Cardiovasc Interv.

[34] Thakkar B, Patel A, Mohamad B (2016). Transcatheter aortic valve replacement versus surgical aortic valve replacement in patients with cirrhosis. Catheter Cardiovasc Interv.

[35] Stefanini GG, Stortecky S, Cao D (2014). Coronary artery disease severity and aortic stenosis: clinical outcomes according to SYNTAX score in patients undergoing transcatheter aortic valve implantation. Eur Heart J.

[36] Thyregod HG, Steinbrüchel DA, Ihlemann N (2015). Transcatheter versus surgical aortic valve replacement in patients with severe aortic valve stenosis: 1-year results from the all-comers NOTION randomized clinical trial. J Am Coll Cardiol.

[37] Bekeredjian R, Szabo G, Balaban U (2019). Patients at low surgical risk as defined by the Society of Thoracic Surgeons Score undergoing isolated interventional or surgical aortic valve implantation: in-hospital data and 1-year results from the German Aortic Valve Registry (GARY). Eur Heart J.

[38] Siontis GC, Overtchouk P, Cahill TJ (2019). Transcatheter aortic valve implantation vs. surgical aortic valve replacement for treatment of symptomatic severe aortic stenosis: an updated meta-analysis. Eur Heart J.

