# Supplementary material for: Clinical outcomes of patients with hepatic insufficiency undergoing transcatheter aortic valve implantation: a systematic review and meta-analysis
Source: BMC Cardiovasc Disord. 2022 Feb 23;22:67. doi: 10.1186/s12872-022-02510-2 (PMC8864911; doi:10.1186/s12872-022-02510-2)
Supplement: Supplementary file 1 — Additional file 1. Table S1. PubMed search strategy. Table S2. Study quality of the included studies (influence of hepatic insufficiency on the postoperative outcome of TAVI). Table S3. Study quality of the included studies (patients with hepatic insufficiency undergoing TAVI verus SAVR). Table S4. GRADE quality assessment of all outcomes. Table S5. The original data of in-hospital mortality after TAVI versus SAVR in patients with hepatic insufficiency. Table S6. The original data of the occurrence rate of blood transfusions after TAVI versus SAVR in patients with hepatic insufficiency. Table S7. The original data of the occurrence rate of acute kidney injury after TAVI versus SAVR in patients with hepatic insufficiency. [file 12872_2022_2510_MOESM1_ESM.docx]

**Supplementary table 1 PubMed search strategy**

#1 Hepatic insufficiency [Mesh]

#2 Hepatic insufficiency OR Liver insufficiency [Title/Abstract]

#3 #1 OR #2

#4 Liver diseases [Mesh]

#5 Liver diseases OR Liver disease OR Liver dysfunction OR Liver dysfunctions [Title/Abstract]

#6 #4 OR #5

#7 Liver cirrhosis [Mesh]

#8 Hepatic cirrhosis OR Liver fibrosis [Title/Abstract]

#9 #7 OR #8

#10 #3 OR #6 OR #9

#11 **Transcatheter aortic valve replacement [Mesh]**

#12 **Transcatheter aortic valve replacement OR Transcatheter aortic valve implantation** OR **TAVR OR TAVI [Title/Abstract]**

#13 #10 OR #11

#14 Aortic Valve Stenosis [Mesh]

#15 Aortic Valve Stenosis OR Aortic Valve Stenoses OR Aortic Stenosis

#16 #13 OR #14

#17 Aortic Valve Insufficiency **[**Mesh**]**

#18 Aortic Valve Incompetence OR Aortic Incompetence OR Aortic Regurgitation OR Aortic Valve Insufficiency

#19 #17 OR #18

#19 Aortic Valve [Mesh]

#20 Aortic Valves OR Aortic Valve [Title/Abstract]

#21 #19 OR #20

#22 #13 OR #16 OR #19 OR #21

#23 #10 AND #22

**Supplementary table 2** Study quality of the included studies.

| Author | Representativeness  of the  exposed cohort | Selection of the non -exposed cohort | Ascertainment of exposure | Demonstration that outcome of interest was not present at start of study | Comparability | Assessment of outcome | follow-up long enough for outcomes to occur | Adequacy of follow up of cohorts | Total scores |
| --- | --- | --- | --- | --- | --- | --- | --- | --- | --- |
| Alqahtani2017 | ★ | ★ | ★ | ★ | ★★ | ★ | ☆ | ★ | 8 |
| Dhoble 2017 | ★ | ★ | ★ | ★ | ★★ | ★ | ☆ | ★ | 8 |
| Greason 2013 | ★ | ★ | ☆ | ★ | ★★ | ★ | ★ | ★ | 8 |
| Khan 2020 | ★ | ★ | ★ | ★ | ★★ | ★ | ☆ | ★ | 8 |
| Peeraphatdit2020 | ★ | ★ | ☆ | ★ | ☆☆ | ★ | ★ | ★ | 6 |
| Seppelt 2020 | ★ | ★ | ★ | ★ | ☆☆ | ★ | ★ | ★ | 7 |
| Thakkar 2015 | ☆ | ★ | ★ | ★ | ★★ | ★ | ☆ | ★ | 7 |
| Lee 2021 | ★ | ★ | ★ | ★ | ★★ | ★ | ☆ | ★ | 8 |

**Supplementary table 3** Study quality of the included studies.

| Author | Is the case definition adequate? | Representativeness of the cases | Selection of Controls | Definition of Controls | Comparability | Ascertainment of exposure | Same method of ascertainment for cases and controls | Non-Response rate | Total scores |
| --- | --- | --- | --- | --- | --- | --- | --- | --- | --- |
| Beohar 2014 | ★ | ★ | ★ | ★ | ☆☆ | ★ | ★ | ☆ | 6 |
| Beohar 2016 | ★ | ★ | ★ | ★ | ☆☆ | ★ | ★ | ☆ | 6 |
| Elbadawi 2019 | ★ | ★ | ★ | ★ | ★★ | ★ | ★ | ☆ | 7 |
| Krittanawong2020 | ★ | ★ | ★ | ★ | ☆☆ | ★ | ★ | ☆ | 6 |
| Lantelme 2020 | ★ | ★ | ★ | ★ | ☆☆ | ★ | ★ | ☆ | 6 |
| Schymik 2015 | ★ | ★ | ★ | ★ | ☆☆ | ★ | ★ | ☆ | 6 |
| Thomas 2011 | ★ | ★ | ★ | ★ | ☆☆ | ★ | ★ | ☆ | 6 |
| Thourani 2016 | ★ | ★ | ★ | ★ | ☆☆ | ★ | ★ | ☆ | 6 |
| Tirado-Conte2018 | ★ | ★ | ★ | ★ | ★★ | ★ | ★ | ☆ | 8 |
| Ullah 2020 | ★ | ★ | ★ | ★ | ☆☆ | ★ | ★ | ☆ | 6 |
| Wendler 2013 | ★ | ★ | ★ | ★ | ☆☆ | ★ | ★ | ☆ | 6 |
| Wendt 2017 | ★ | ★ | ★ | ★ | ★★ | ★ | ★ | ☆ | 8 |
| Yassin 2018 | ★ | ★ | ★ | ★ | ★★ | ★ | ★ | ☆ | 8 |

**Supplementary table 4** GRADE quality assessment of all outcomes

| Outcomes (number of studies) | Risk of bias | Inconsistency | Indirectness | Imprecision | Publication bias | Relative effect (95% CI) | Quality of the evidence |
| --- | --- | --- | --- | --- | --- | --- | --- |
| The first analysis | | | | | | | |
| Short-term mortality (5) | Not serious | Serious | Not serious | Not serious | Serious | OR=1.88 (1.38-2.58) | ◯◯◯ Very low |
| 1-2 years mortality (8) | Not serious | Not serious | Not serious | Not serious | Serious | HR=1.64 (1.42-1.89) | ◯◯◯ Very low |
| The second analysis | | | | | | | |
| In-hospital mortality (7) | Not serious | Not serious | Not serious | Not serious | Not serious | OR=0.46 (0.27-0.81) | ◯◯ Low |
| Blood transfusions (5) | Not serious | Not serious | Not serious | Not serious | Not serious | OR=0.29 (0.22-0.38) | ◯◯ Low |
| Acute kidney injury (4) | Not serious | Not serious | Not serious | Not serious | Not serious | OR=0.55 (0.33-0.91) | ◯◯ Low |

**Supplementary table 5** The original data of in-hospital mortality after TAVI versus SAVR in patients with hepatic insufficiency.

| ID | Author and year | TAVR | | SAVR | |
| --- | --- | --- | --- | --- | --- |
|  |  | Events | Total | Events | Total |
| 1 | Thakkar 2015 | 2 | 30 | 2 | 30 |
| 2 | Greason 2013 | 0 | 6 | 2 | 12 |
| 3 | Alqahtani 2017 | 11 | 134 | 25 | 134 |
| 4 | Dhoble 2017 | 9 | 126 | 7 | 157 |
| 5 | Peeraphatdit 2020 | 1 | 55 | 1 | 50 |
| 6 | Khan  2020 | 15 | 273 | 78 | 404 |
| 7 | Seppelt 2019 | 7 | 43 | 9 | 42 |

**Supplementary table 6** The original data of the occurrence rate of blood transfusions after TAVI versus SAVR in patients with hepatic insufficiency.

| ID | Author and year | TAVR | | SAVR | |
| --- | --- | --- | --- | --- | --- |
|  |  | Events | Total | Events | Total |
| 1 | Thakkar 2015 | 9 | 30 | 17 | 30 |
| 2 | Alqahtani 2017 | 35 | 134 | 63 | 134 |
| 3 | Dhoble 2017 | 12 | 55 | 32 | 55 |
| 4 | Peeraphatdit 2020 | 13 | 55 | 33 | 50 |
| 5 | Khan  2020 | 55 | 273 | 185 | 404 |

**Supplementary table 7** The original data of the occurrence rate of acute kidney injury after TAVI versus SAVR in patients with hepatic insufficiency.

| ID | Author and year | TAVR | | SAVR | |
| --- | --- | --- | --- | --- | --- |
|  |  | Events | Total | Events | Total |
| 1 | Thakkar 2015 | 6 | 30 | 9 | 30 |
| 2 | Alqahtani 2017 | 11 | 134 | 19 | 134 |
| 3 | Peeraphatdit 2020 | 2 | 55 | 4 | 50 |
| 4 | Seppelt 2019 | 12 | 43 | 17 | 42 |
